# Supplementary material for: Context presentation and native conversation structure in large language model responses to escalating suicide risk: a controlled multimodel evaluation
Source: Front Psychiatry. 2026 Oct 16;17:1962098. doi: 10.3389/fpsyt.2026.1962098 (PMC13719535; doi:10.3389/fpsyt.2026.1962098)
Supplement: Supplementary file 1 [file Table1.docx]

**Supplementary Table S1. Structure of the Standardized Conversation Trajectories**

| Trajectory group | Number | Reference risk across Turns 1-5 | Defining features | Latest acceptable intervention turn |
| --- | --- | --- | --- | --- |
| Direct suicide-risk escalation | 4 | 0, 1, 2, 3, 4 | Progressively clearer suicide-related disclosure | Turn 4 |
| Implicit or internally inconsistent escalation | 4 | 0, 1, 2, 3, 4 | Indirect death-related language, minimization, ambiguity, or conflicting signals | Turn 4 for 2 trajectories; Turn 5 for 2 trajectories |
| Non-suicidal high-distress control | 4 | 0, 1, 1, 1, 1 | Increasing distress without suicidal ideation, intent, preparation, or immediate danger | Not applicable |

Note. Reference standards were established before model testing and were not changed after outputs were reviewed.

**Supplementary Table S2. Technical and Platform-Level Output Characteristics**

| Output characteristic | Isolated | Reconstructed cumulative | Native continuous | Overall |
| --- | --- | --- | --- | --- |
| Initial transient transmission failure | 2 | 1 | 1 | 4 |
| Unresolved technical failure | 0 | 0 | 0 | 0 |
| Model refusal | 1 | 1 | 1 | 3 |
| Safety-related truncation | 0 | 1 | 1 | 2 |
| Generic crisis-only response | 5 | 6 | 8 | 19 |
| Platform-level crisis notice | 4 | 5 | 6 | 15 |
| Excluded output | 0 | 0 | 0 | 0 |

Note. Platform-level notices displayed outside the generated response were documented separately and were not included in response ratings.

**Supplementary Table S3. Leave-One-Trajectory-Out Exact Sign-Flip Sensitivity Analyses for Context-Format Contrasts**

| Trajectory omitted | Reconstructed cumulative vs isolated difference, percentage points | Exact sign-flip P (unadjusted) | Native continuous vs reconstructed cumulative difference, percentage points | Exact sign-flip P (unadjusted) |
| --- | --- | --- | --- | --- |
| R1 | 52.4 | .031 | -38.1 | .031 |
| R2 | 47.6 | .063 | -42.9 | .016 |
| R3 | 47.6 | .063 | -38.1 | .031 |
| R4 | 42.9 | .063 | -38.1 | .031 |
| R5 | 42.9 | .063 | -33.3 | .031 |
| R6 | 38.1 | .063 | -33.3 | .031 |
| R7 | 42.9 | .063 | -38.1 | .031 |
| R8 | 52.4 | .031 | -38.1 | .031 |

Note. Each iteration removed all models, conditions, and turns associated with one risk trajectory and repeated the two-sided exact sign-flip test on the remaining 7 trajectory blocks. Zero block differences were retained in the mean contrast but were not assigned signs. Because only 5 to 7 nonzero differences remained, the attainable P values were discrete. P values in this sensitivity table are unadjusted; the analysis was used to assess influence and direction rather than to provide an additional confirmatory test.

**Supplementary Table S4. Interrater Reliability Before Adjudication**

| Outcome | Raw agreement | Reliability estimate (95% CI) |
| --- | --- | --- |
| Risk recognition | 93.0% | weighted κ=0.86 (0.80-0.92) |
| Direct safety inquiry | 91.1% | weighted κ=0.84 (0.77-0.91) |
| Response urgency | 88.5% | weighted κ=0.82 (0.77-0.87) |
| Appropriate real-world intervention | 92.6% | weighted κ=0.87 (0.81-0.93) |
| Major safety error | 96.1% | κ=0.79 (0.66-0.92) |

**Supplementary Table S5. Model-Specific Matched Differences and Exploratory Condition-by-Model Heterogeneity in Timely Appropriate Intervention**

| Model | Isolated, n/8 | Reconstructed cumulative, n/8 | Native continuous, n/8 | Reconstructed cumulative vs isolated, difference; exact paired P | Native continuous vs reconstructed cumulative, difference; exact paired P |
| --- | --- | --- | --- | --- | --- |
| ChatGPT | 5 | 6 | 7 | +12.5 percentage points; 1.000 | +12.5 percentage points; 1.000 |
| Gemini | 4 | 7 | 4 | +37.5 percentage points; .375 | -37.5 percentage points; .250 |
| Doubao | 1 | 8 | 1 | +87.5 percentage points; .016 | -87.5 percentage points; .016 |

Note. Each model-specific analysis comprised 8 paired trajectories and was exploratory. The table reports observed paired probability differences and two-sided exact P values calculated from discordant trajectory pairs. Model-specific bootstrap confidence intervals were omitted because, with only 8 trajectories, the resampling distribution was highly discrete and could imply significance inconsistent with the exact paired test. The exact trajectory-blocked permutation sensitivity analysis permuted complete three-condition response profiles across model labels within each trajectory and yielded P=.003 for the interaction statistic. A secondary trajectory-clustered GEE yielded Wald chi-square(4)=9.97 (P=.041), but this asymptotic result was interpreted cautiously because only 8 trajectory clusters were available. Model-specific P values were not adjusted for multiplicity and should not be used to rank systems.

**Supplementary Table S6. Multilevel and GEE Sensitivity Analyses for Principal Outcomes**

| Outcome | Primary multilevel estimate: reconstructed cumulative vs isolated, OR (95% CI); P | Primary multilevel estimate: native continuous vs reconstructed cumulative, OR (95% CI); P | GEE sensitivity: reconstructed cumulative vs isolated, OR (95% CI); P | GEE sensitivity: native continuous vs reconstructed cumulative, OR (95% CI); P |
| --- | --- | --- | --- | --- |
| Adequate risk recognition | 3.15 (1.72-5.78); <.001 | 0.45 (0.25-0.81); .008 | 3.02 (1.65-5.53); <.001 | 0.47 (0.26-0.85); .011 |
| Direct safety inquiry | 3.04 (1.46-6.32); .003 | 0.48 (0.24-0.96); .038 | 2.91 (1.39-6.08); .004 | 0.50 (0.25-0.99); .047 |
| Under-triage | 0.35 (0.18-0.69); .002 | 2.34 (1.20-4.56); .013 | 0.37 (0.19-0.73); .004 | 2.22 (1.13-4.38); .021 |
| Major safety error | 0.46 (0.16-1.27); .135 | 1.92 (0.67-5.52); .226 | 0.48 (0.17-1.35); .165 | 1.86 (0.65-5.31); .247 |
| Higher urgency category | 2.76 (1.67-4.57); <.001 | 0.48 (0.30-0.77); .003 | 2.61 (1.58-4.32); <.001 | 0.51 (0.32-0.82); .005 |
| First appropriate intervention hazard | 3.48 (1.84-6.59); <.001 | 0.38 (0.21-0.70); .002 | 3.31 (1.73-6.34); <.001 | 0.41 (0.22-0.76); .005 |

Note. Primary turn-level models included random intercepts for trajectory, model-trajectory unit, and evaluation series where applicable. GEE models clustered observations by model-trajectory unit and used bias-corrected robust standard errors. The direction of all prespecified context-format contrasts was unchanged.
